# Polyamines Involved in Regulating Self-Incompatibility in Apple

**DOI:** 10.3390/genes12111797

**Published:** 2021-11-15

**Authors:** Jie Yu, Baoan Wang, Wenqi Fan, Songbo Fan, Ya Xu, Chunsheng Liu, Tianxing Lv, Wanda Liu, Ling Wu, Linfeng Xian, Tianzhong Li

**Affiliations:** 1 College of Horticulture, China Agricultural University, Beijing 100193, China; yujie7616@163.com (J.Y.); poanwang@163.com (B.W.); fanwenqi639@163.com (W.F.); fansongbo1998@163.com (S.F.); xuya1270@163.com (Y.X.); liuchunsheng2016@163.com (C.L.); lingwu166@163.com (L.W.); xianlinfeng0@163.com (L.X.); 2 Institute of Pomology, Liaoning Academy of Agricultural Sciences, Yingkou 115009, China; ltxssrlcy@163.com; 3 Horticultural Branch, Heilongjiang Academy of Agricultural Sciences, Harbin 150000, China; haaslwd@126.com

**Keywords:** polyamines, apple, self-incompatibility, S-RNase, pollen tube

## Abstract

Apple exhibits typical gametophytic self-incompatibility, in which self-S-RNase can arrest pollen tube growth, leading to failure of fertilization. To date, there have been few studies on how to resist the toxicity of self-S-RNase. In this study, pollen tube polyamines were found to respond to self-S-RNase and help pollen tubes defend against self-S-RNase. In particular, the contents of putrescine, spermidine, and spermine in the pollen tube treated with self-S-RNase were substantially lower than those treated with non-self-S-RNase. Further analysis of gene expression of key enzymes in the synthesis and degradation pathways of polyamines found that the expression of *DIAMINE OXIDASE 4* (*MdDAO4*) as well as several polyamine oxidases such as *POLYAMINE OXIDASES 3* (*MdPAO3*), *POLYAMINE OXIDASES 4* (*MdPAO4*), and *POLYAMINE OXIDASES 6* (*MdPAO6*) were significantly up-regulated under self-S-RNase treatment, resulting in the reduction of polyamines. Silencing *MdPAO6* in pollen tubes alleviates the inhibitory effect of self-S-RNase on pollen tube growth. In addition, exogenous polyamines also enhance pollen tube resistance to self-S-RNase. Transcriptome sequencing data found that polyamines may communicate with S-RNase through the calcium signal pathway, thereby regulating the growth of the pollen tubes. To summarize, our results suggested that polyamines responded to the self-incompatibility reaction and could enhance pollen tube tolerance to S-RNase, thus providing a potential way to break self-incompatibility in apple.

## 1. Introduction

Apple needs cross-pollination to guarantee yield in production, as it exhibits S-RNase mediated self-incompatibility. In the process of self-incompatibility, S-RNase secreted from the styles enters into the pollen tube with the assistance of the ABC transporter F family member 3-like protein (ABCF) protein, and then Self S-RNase is recognized by S-locus F-box brothers (SFBB) and labeled with ubiquitin [1]. After self-/non-self-discrimination, non-self S-RNase will be ubiquitinated and degraded through the 26S protease pathway, while self S-RNase continues to remain in the cytoplasm, playing a toxic effect, capturing the elongation of the pollen tube [2]. Previous studies found that the pollen tube of apple responded to the invasion of S-RNase before self/non-self-recognition, subsequently activating a defensive response mediated by the jasmonic acid signal and temporarily delaying the toxicity of S-RNase in the pollen tube, thus protecting the pollen tube from S-RNase during self/non-self-recognition [3]. Self S-RNase inhibits the elongation of pollen tubes by inhibiting tRNA aminoacylation, protein synthesis, and breaking the cleavage dynamics of microfilaments [4]. So far, no research has shown whether the pollen tube is capable of resisting self S-RNase after self/non-self-discrimination.

To date, it has been reported that several ways of breaking self-incompatibility can be applied. In *Raphanus sativus*, it has been demonstrated that treating flowering branches at 50 °C for 25 min can significantly improve the self-fertilized fruit setting rate [5]. In *Lilium longiflorum* Thunb, applying stamen extract on the stigma could also break self-incompatibility to a certain extent [6]. In addition, Williams et al., have also proven that the self-fertilized seed setting rate of incompatible apples could be improved when self-pollen was mixed with methanol inactivating non-self pollen [7].

Polyamines are physiologically active substances, which widely exist in eukaryotes and prokaryotes [8]. In higher plants, polyamines mainly exist in a free state. Putrescine (Put), spermidine (Spd), and spermine (Spm) are the main polyamines in plants. They are involved in the regulation of a variety of growth and development processes [9]. For instance, plant polyamines play a vital role in cell division, embryogenesis, and root growth [10]. Moreover, polyamines are also involved in the response to both biological and abiotic stresses, such as improving the drought resistance of rice [11], as well as participating in the process of resistance to pathogens [12]. In Pyrus sp., studies have found that polyamines are related to the reaction of self-incompatibility. In particular, the levels of three polyamines (Put, Spd, and Spm) in incompatible reactions were substantially lower than that of the compatible reaction [13]. Furthermore, it was found that application of spermidine in vitro is conducive to improving the fruit setting rate in apples [14], whether the metabolism of polyamines responds to S-RNase and whether exogenous polyamine treatment can break the self-incompatibility remains to be explored.

There are two currently recognized polyamine anabolic pathways. One is the ornithine pathway, in which ornithine generates putrescine under the catalysis of ornithine dehydrogenase (ODC), and the other is the arginine pathway, in which arginine generates herring spermine under the catalysis of arginine dehydrogenase (ADC), and the herring spermine is hydrolyzed to putrescine [12,14,15]. Spermidine and spermine are formed by putrescine plus aminopropyl both on one and two sides. SAMDC catalyzes S-adenosylmethionine decarboxylation (SAM) to generate aminopropyl. Putrescine generates spermidine under the action of spermidine synthase (SPDS), and spermidine generates spermine under the action of spermine synthase (SPMS). The degradation of polyamines is catalyzed by oxidase. In the polyamine metabolic pathway, it is mainly catalyzed by diamine oxidase (DAO) and polyamine oxidase (PAO). Diamine oxidase catalyzes the decomposition of putrescine to 4-aminobutanal, and H_2_O_2._ Polyamine oxidase is responsible for decomposing spermidine and spermine to produce the corresponding 1,3-diaminopropane, amino aldehyde and H_2_O_2._ [16]. These genes in the polyamine metabolism pathway play an important role in pollen germination and pollination. Some studies have found that the decrease of SAMDC activity is the main reason for the inhibition of tomato pollen germination caused by high temperature in rice [17]; it has been found that diamine oxidase plays an important role in rice anther cracking, pollen fertility, and seed generation. Silencing diamine oxidase leads to abnormal anther cracking and reduced pollen fertility [18]. In *Arabidopsis* mutant Atpao3, pollen could not be induced by spermidine (Spd) to open the calcium channels, which showed that pollen length and seed setting rate decreased [19]. Whether genes in the polyamine synthesis pathway participate in self-incompatibility has seldom been studied. In our study, we explore the molecular mechanism of polyamines participating in self incompatibility and provide gene resources for creating self-compatible apple.

## 2. Materials and Methods

### 2.1. Plant Material

*Malus domestica* cv. ‘Ralls Janet’ (S1S2) is a common apple cultivar. The studied trees are grown in Shangzhuang Experiment Station, Haidian, Beijing, China. Leaves, pollen, styles, ovary, sepals, filaments, and petals were collected and stored at −80 °C until further use. The pollen was hydrated in liquid medium containing 10% (*w*/*v*) sucrose, 0.01% (*w*/*v*) H_3_BO_3_, and 0.015% (*w*/*v*) CaCl_2_. The coding sequences (CDSs) of S1-, S2-, S3-, and S9-RNase were cloned into the pEASY-E1(Transgene, Beijing, China) expression vector and expressed as His-fusion proteins in *Escherichia coli* strain BL21 (DE3) (Transgene, Beijing, China). Expression and purification of the RNase proteins were performed as described by Meng et al. [1].

### 2.2. Pollen Treated with Self- and Non-Self-S-RNase

After 30 min of hydration, the pollen in the deep freezer was cultured in the liquid medium with 25 μg mL^−1^ self-S-RNase and non-self-S-RNase. S-RNase was expressed and purified by Prokaryotic Expression as described by Meng, et al. [20] and buffer added respectively. After culturing in a dark humid environment at 25 °C for 0 min, 30 min, 60 min, 90 min, and 120 min, the pollen liquid medium was centrifuged to collect the pollen, and then the pollen was washed with PBS buffer (pH = 7.0) five times and the pollen stored for subsequent experiments.

### 2.3. Antisense Oligonucleotide Transfection

We designed a phosphorothioated antisense oligodeoxynucleotide (as-ODN) based on the specific region of the coding sequence, to down-regulate gene expression. Sense oligodeoxynucleotide(s-ODN) was also used. The oligodeoxynucleotide sequences used in this study are listed in the Supplemental Table S4. Pollen was hydrated in the liquid medium for 30 min, and then the s-ODN mixture or as-ODN mixture should be cultured for 15 min. According to the requirements of the experiment, self-S-RNase, non-self-S-RNase, or buffer should be added to the pollen liquid medium. After the pollen tube was hydrated, MdDAO4, MdPAO3, MdPAO4, MdPAO6 antisense oligonucleotide strands, and sense oligonucleotide strands were added to the medium, and the pollen tubes were incubated for 15 min. Then we added self- and non-self-S-RNase to the cultured pollen tubes. Pollen tubes treated with a blank medium served as a control. We collected the pollen tubes after 90 min, measured the length of the pollen tubes, and tested the polyamine content in the pollen tubes for seven different treatments, including only medium. Sense oligonucleotide strands or antisense oligonucleotide strands were added, and self-S-RNase or non-self-S-RNase was added after the sense oligonucleotide strands or antisense oligonucleotide strands had been treated. 

The mixture used for transfection included 2 mL pollen germination medium, 20 μL oligodeoxynucleotide (100 μM), 280 μL cytofectin buffer, and 40 μL cytofectin (Lipofectamine 3000; Invitrogen, Waltham, MA, USA), and it was pre-mixed and cultured at 37 °C for 15 min.

### 2.4. HPLC Analysis of Polyamines

Polyamine analysis was carried out as described by Flores [21], but with some modifications. An amount of 0.5 g anthers was weighed and the pollen collected with PBS buffer (0.1 M Na_2_HPO_4_ and 0.1 M NaH_2_PO_4_, pH = 7.0), then the pollen was mixed in 1 mL of extraction solution (5% HClO_4_) and stirred into the homogenate. The solution was placed in an ice bath for 30 min and then centrifuged at 4 °C for 30 min; 1 mL of supernatant was removed and 7 μL of benzoyl chloride and 1 mL of 2 mol/L sodium hydroxide were added. After being vortexed for 30 s, it was placed in a 37 °C water bath for 30 min, 2 mL of saturated sodium hydroxide was added to terminate the reaction, and 1 mL of ether was added for extraction; it was then centrifuged at 15,000 rpm for 30 min, 1 mL of supernatant was removed, and a vacuum dryer was used to volatilize the ether. All reagents and samples were required to be filtered with a 0.22 μm filter membrane.

HPLC analysis was performed using a Waters liquid chromatography system (Milford, MA, USA). Polyamines were separated and analyzed via HPLC on a C18 column (4.6 × 100 mm, 3.5-Micron) with a mobile phase of methanol/double distilled water (64/36 *v*/*v*) at a flow rate of 0.7 mL/min. Absorbance was measured at 230 nm. Samples were tested using three biological replicates.

### 2.5. RNA Extraction and qPCR Assay

RNA was extracted from the pollen using an RN53 EASYspin Plus Complex Plant RNA extraction kit (Aidlab, China). cDNAs were synthesized from 0.5 μg of total RNA using ReverTra Ace™ qPCR RT Master Mix with gDNA Remover (Toyobo, Japan). For quantitative RT-PCR analysis, SuperReal PreMix Plus (SYBR Green) was used (Beijing, China). The qRT-PCR was conducted with three biological replicates, and each sample was analyzed at least three times and normalized using MdACTIN as an internal control. Transcription levels were assessed using the 2^−ΔΔC^_T_ method [22].

Most genes related to polyamines synthetic and degradation enzymes in plants have been identified, including ornithine decarboxylase (ODC), arginine decarboxylase (ADC), spermidine synthase (SPDS), spermine synthase (SPMS), S-adenosylmethionine decarboxylase (SAMDC), diamine oxidase (DAO), and polyamine oxidase (PAO). These important genes have many members, we identified a total of 2 ODC, 2 ADC, 4 SPDS, 4 SPMS, 6 SAMDC, 7 DAO, and 6 PAO genes from the apple genome, as shown in Table S1. The total RNA was extracted from seven different apple organs including leaves, pollen, styles, ovary, sepals, filaments, and petals. Expression tissues of the genes were then detected using qRT-PCR. Genetic structure and tissue specific expression analysis are shown in Figure S2. All primers are shown in the supplementary data Table S2. 

### 2.6. Exogenous Polyamine Treatments

Pollen was cultured in the liquid medium for 30 min and after the pollen was hydrated, 25 μg ml^−1^ self- or non-self-S-RNase was added. The polyamines (Sigma, Burlington, MA, USA) were dissolved by ddH_2_O to 2 mmol/L, then the polyamine solution added to the pollen medium to a final concentration of 0.005 mmol/L, 0.025 mmol/L, 0.05 mmol/L, 0.25 mmol/L, 0.5 mmol/L, 1 mmol/L, and the polyamines mixed gently. Pictures were taken and the length of the pollen tube was measured after 90 min incubation.

### 2.7. RNA Extraction, Library Preparation and RNA-Seq

Total RNA was extracted from the pollen using an RN53 EASYspin Plus Complex Plant RNA extraction kit (Aidlab, China). Agarose gel electrophoresis was used to analyze the degree of RNA degradation. The purity of RNA was determined by Nanodrop (OD260/280 ratio) and the RNA library preparations and sequencing by Novogene Bioinformatics Technology Co., Ltd. (Beijing, China) on an Illumina HiSeqTM PE150 platform. The total number of raw reads obtained from each sample ranged from 32.17 million to 50.14 million. After removing the adapter and low-quality reads. The clean reads were mapped to the apple reference genome (https://www.rosaceae.org/species/malus/malus_x_domestica/genome_GDDH13_v1.1, accessed on 10 October 2021), using TopHat software. For gene expression analyses, FPKM values were calculated using RESM software [23]. The differentially expressed genes (DEGs) were analyzed using DESeq software with the following criteria: *p*-value < 0.05 and | log2(Fold Change) | ≥ 0.58 [24]. Three biological replicates were used for pollen treated with 0.25 mmol/L spermidine and untreated pollen under the self-incompatibility reaction. The DEGs were also analyzed using Gene Ontology (GO; www.geneontology.org, accessed on 20 August 2021) and the Kyoto Encyclopedia of Genes and Genomes (KEGG; www.genome.jp/kegg, accessed on 20 August 2021) tools. The read numbers were transformed to FPKM (Fragments Per Kilobase of transcript sequence per Millions of base pairs sequenced) value for gene expression quantification. We randomly selected 12 genes from the differentially expressed genes and verified them by RT-PCR. All primers are shown in the supplementary data Table S2.

### 2.8. Aniline Blue Staining and Fluorescence Microscopy

After pollination, pistils were collected and stored in FAA fixed solution (5% formalin, 5% acetic acid, and 63% ethanol). As described by Meng et al., the pollinating style was stained with aniline blue and observed using fluorescence. A 405-nm diode was used for excitation, and the samples were analyzed using a confocal microscope.

## 3. Results

### 3.1. Polyamine Level in Pollen Treated with Self- or Non-Self-S-RNase Differs

**Figure 1:**
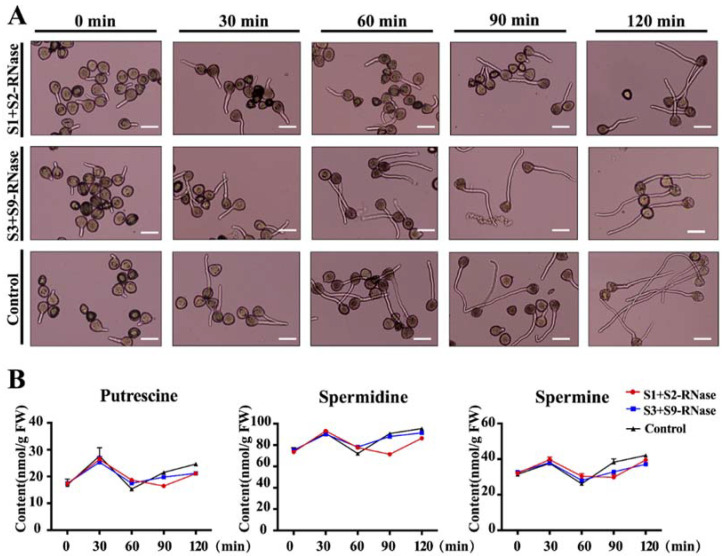
Effects of self-S-RNase on pollen tube length and polyamine contents in pollen tubes. (**A**) After hydration, the germination status of the pollen tube was observed at 0, 30 min, 60 min, 90 min, 120 min after self- and non-self-S-RNase treatment, the control represents the untreated pollen tubes, and the scale bar represents 15 μm. (**B**) The content of putrescine, spermidine, and spermine: self-S-RNase, non-self S-RNase and control. Values are means of three biological replicates.

To determine whether the metabolism of polyamines is related to self- incompatibility, we established treated ‘Ralls Janet’(S1S2) pollen with self-S-RNase (S1 and S2-RNase), non-self-S-RNase (S3 and S9-RNase), and control (protein buffer), respectively. The result showed that self-S-RNase treated pollen tube growth was strongly inhibited and showed the greatest difference from the non-self-S-RNase treated ones at 90 min (Figure 1A). Statistical results of pollen tube length (n = 3, with 50 pollen grains per replication) are shown in the supplementary data (Figure S1). We next detected the polyamine content in both self- and non-self-S-RNase treated pollen tubes. Three core polyamines (putrescine, spermidine, spermine) were identified in the pollen tubes at five stages after being treated, as shown in Figure 1B. The contents of spermine and spermidine in the pollen tube were 24.84–41.13 nmol/g FW and 71.32–93.42 nmol/g FW, higher than that of putrescine, which was 15.15–29.45 nmol/g FW. After added self-S-RNase, non-self-S-RNase, and control, the polyamines in the pollen tubes increased and then decreased. The difference in the contents of the three polyamines was most significant at 90 min after treatment, and the self-S-RNase induced pollen tubes contained the lowest number of polyamines (Figure 1B).

### 3.2. Expression Analysis Revealed the Polyamine Degradation Pathway Associated with Self-Incompatibility 

**Figure 2:**
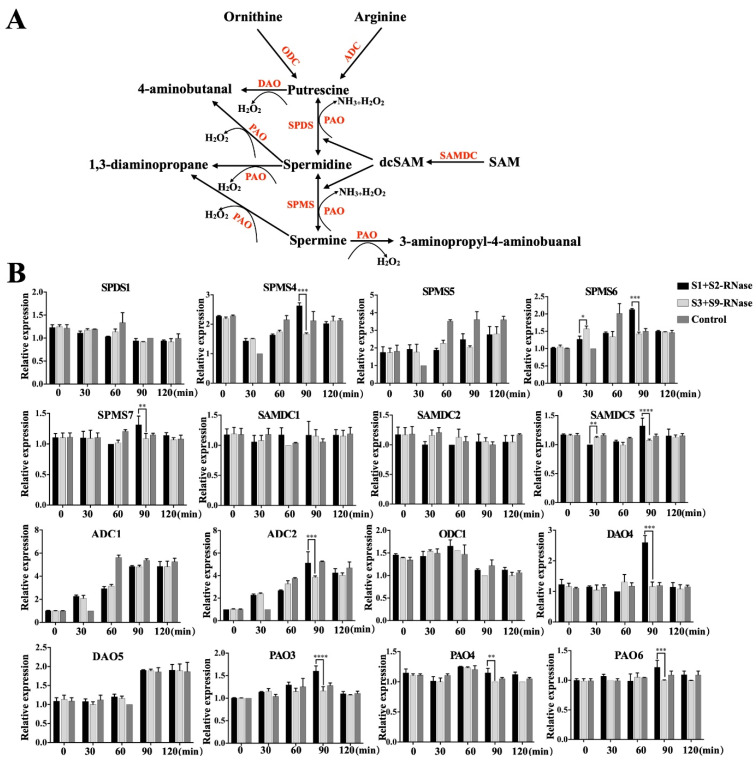
Expression of key genes in the polyamine biosynthetic and oxidative pathway. (**A**) A schematic diagram on synthesis and degradation of polyamines. (**B**) The relative expression of the genes on the polyamine pathway: ODC, ornithine decarboxylase; ADC, arginine decarboxylase; SAMDC, S-Adenosyl-L-Methionine Decarboxylase; SPDS, spermidine synthase; SPMS, spermine synthase; DAO, diamine oxidase; PAO, polyamine oxidase. Data are means ± SD of three independent experiments. Bars indicate SD. Asterisks indicate significant differences between self-S-RNase and non-S-RNase treated. *, *p* < 0.05; **, *p* < 0.01; ***, *p* < 0.001; ****, *p* < 0.0001 by Tukey’s multiple comparisons test. The control represents buffer treated pollen tubes.

In order to determine the genes involved in the polyamine metabolic pathway responsible for self-incompatibility, we treated ‘Ralls Janet’ (S1S2) pollen with self-S-RNase (S1 and S2), non-self-S-RNase (S3 and S9), and control (buffer) respectively. We examined gene expression after treatment at 0 min, 30 min, 60 min, 90 min, and 120 min, and found that many genes on the polyamine metabolic pathway responded to self-incompatibility (Figure 2B). We found oxidize putrescine DAO4, the genes that oxidize spermidine and spermine PAO3, PAO4, and PAO6, the polyamines degradation process, and the related oxidase genes which were of interest due to their association with self-incompatibility.

### 3.3. Silencing MdPAO6 by Transfection with Antisense Oligodeoxynucleotides Weakens the Self-S-RNase-Mediated Inhibition of Pollen Tubes

**Figure 3:**
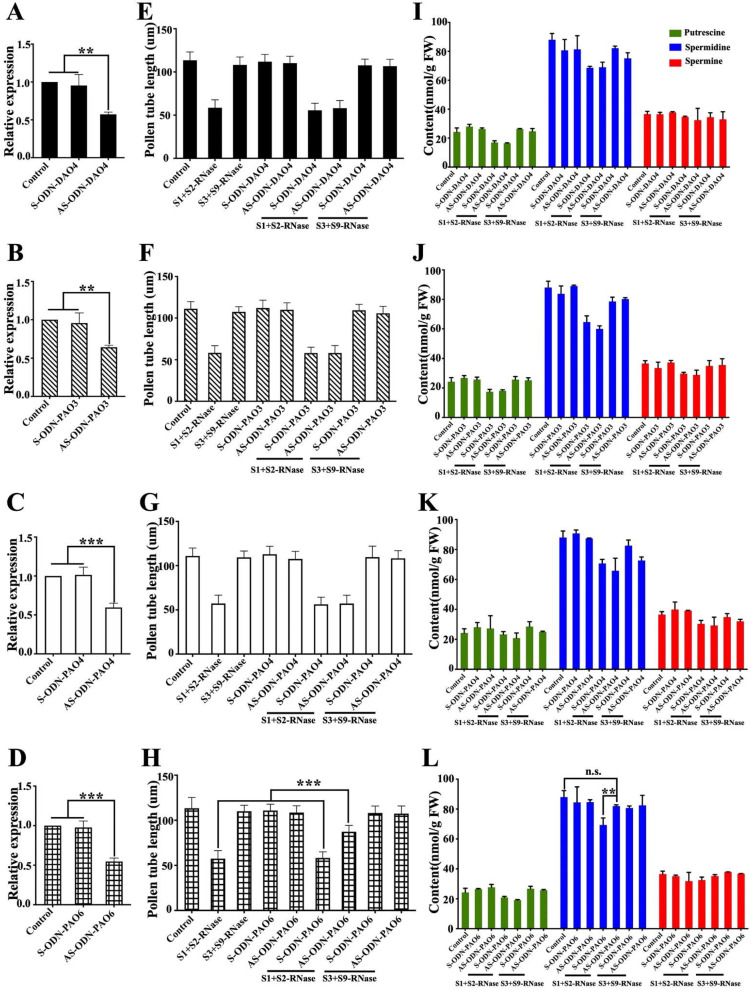
Effect of oxidase genes on pollen tube elongation and polyamine content. (**A**–**D**) Expression levels of *MdDAO4*, *MdPAO3*, *MdPAO4*, and *MdPAO6* in pollen transfected with sense oligonucleotide, antisense oligonucleotide or transfection agent alone (Control) during pollen tube growth medium. (**E**–**H**) Pollen tube growth in response to S-RNase after transfection with sense or antisense oligonucleotide of *MdDAO4*, *MdPAO3*, *MdPAO4*, and *MdPAO6*, The control represents buffer treated pollen tubes (at least 50 pollen tubes per treatment). (**I**–**L**) The content of putrescine (green), spermidine (blue), and spermine (red): control, transfected with sense oligonucleotide, transfected with antisense oligonucleotide, S-RNase treated after being transfected with sense oligonucleotide and transfected with antisense oligonucleotide. Values are means of three biological replicates. Bars indicate SD. Asterisks indicate significant difference between self-S-RNase treated after being transfected with sense oligonucleotide and antisense oligonucleotide by using Tukey’s multiple comparisons test. (** *p* < 0.01; ***, *p* < 0.001 by Tukey’s multiple comparisons test).

Considering the importance of the polyamine degradation pathway and associated genes for pollen tube growth, we explored the potential role of MdDAO4, MdPAO3, MdPAO4, MdPAO6 oxidase genes by antisense oligonucleotide transfection. Transfection of ‘Ralls Janet’ pollen with antisense oligonucleotide decreased the expression of the corresponding four candidate genes (Figure 3A,D,G,J). We found that MdPAO6 antisense nucleotide transfection significantly reduced the inhibition of self-S-RNase in pollen tube growth (Figure 3L). This indicates that MdPAO6 is essential for pollen tubes to respond to self-incompatibility.

We found that the expression of MdPAO6 decreased after antisense oligonucleotide treatment in pollen. After self-S-RNase treatment, the content of spermidine in the pollen tubes increased significantly, and there was no significant difference with untreated pollen tubes.

We speculated a response to self-incompatibility reactions; MdPAO6 gene expression was increased, thus spermidine content decreased in the pollen tubes, eventually leading to pollen tube growth being restrained. When we silenced polyamine oxidase MdPAO6, the spermidine content increased with self-S-RNase treated, thereby breaking the inhibition of pollen tube growth by self-S-RNase.

### 3.4. Exogenous Application of Polyamine Improved Tolerance of Apple Pollen to Self-S-RNase

**Figure 4:**
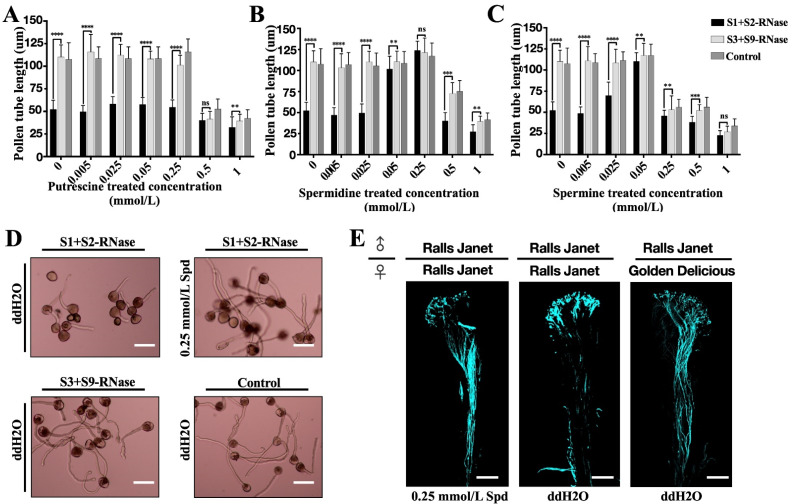
Effects of exogenous polyamines on self-S-RNase tolerance of pollen tubes. Effects of exogenous polyamines on pollen tube elongation. (**A**–**C**) After putrescine, spermidine, and spermine treatment, the effect of S-RNase on pollen tube length. Data show means ± SD of three biological replicates. Per treatment at least 50 pollen tubes. (**D**) Upper: Pollen tube germination after self-S-RNase was added (left), Pollen tube germination after self-S-RNase and 0.25 mmol/L were added (right), Lower: Pollen tube germination after non-self-S-RNase was added (left) and only pollen germinated in the medium (right). (**E**) After 0.25 mmol/L spermidine treated and control (ddH_2_O) treated, aniline blue staining of ‘Ralls Janet’ pollen on ‘Ralls Janet’ pistil and ‘Ralls Janet’ pollen on ‘Golden Delicious’ pistil for 72 h, Scale bars represent 550 μm. **, *p* < 0.01; ***, *p* < 0.001; ****, *p* < 0.0001 by Tukey’s multiple comparisons test.

To explore whether exogenous polyamine can reduce the toxicity of self-S-RNase to pollen, we prepared putrescine, spermidine, and spermine at concentrations of 0.005 mmol/L, 0.025 mmol/L, and 0.05 mmol/L, 0.25 mmol/L, 0.5 mmol/L, 1 mmol/L and added them to pollen medium respectively. It was found that the pollen tube growth was inhibited by the addition of 0.5 mmol/L, 1 mmol/L putrescine or spermidine in the medium, even with or without the addition of non-self-S-RNase. When we added self-S-RNase to the pollen tubes medium, the inhibition of pollen tube growth was strengthened. The same inhibitory effect could be achieved by adding 0.25 mmol/L spermine. After adding 0.05 mmol/L, 0.25 mmol/L spermidine to the pollen medium, it significantly improved the pollen tube’s resistance to self-S-RNase (Figure 4A,B,D). After adding 0.05 mmol/L spermine to the pollen medium, the resistance of the pollen tubes to self-S-RNase was increased and the length of pollen tube germination was increased (Figure 4C). In order to verify whether the exogenous application of spermidine helps to break the self-incompatibility, we applied 0.25 mmol/L spermidine on the stigma of ‘Ralls Janet’, and then took off the style 72 h after pollination with ‘Ralls Janet’. We found that the pollen tube had grown to the bottom of the style. However, the pollen tube had only grown to one-quarter of the style after self-pollinated (Figure 4E).

### 3.5. RNA-Seq Analysis of Spermidine Treated and Control Treated Self-Incompatible Pollen

We used an RNA-Seq approach to explore differential gene expression among the different treatments. The two treatments were pollen treated with 0.25 mmol/L spermidine and untreated pollen under self-incompatibility reaction, respectively. After filtering, the number of clean reads per library ranged from 32.03 million to 49.57 million. Greater than 97% of the clean reads of all samples could be uniquely mapped to the apple genome. A Pearson correlation analysis indicated that gene expression levels between the biological replicates were highly related (R2 > 0.96). The table of sequencing data quality is shown in Table S3. The differentially expressed gene DEGs were identified and filtered for |log2(Fold Change)| > 0.58, *p*-value < 0.05. In the control samples vs. spermidine treated samples, a total of 13,164 non-redundant DEGs was obtained, 50 genes were upregulated and 104 genes were downregulated in the control relative to the spermidine treated samples. Gene ontology (GO) enrichment analysis was performed on DEGs. The Gene ontology (GO) analysis indicated that these genes were involved in diverse biological processes, cellular components, and molecular functions, such as cytoplasm, antioxidant activity, binding, biological regulation, and catalytic activity. Kyoto Encyclopedia of Genes and Genomes (KEGG) pathways were identified to be significant (*p*-value < 0.05) in the differentially expressed genes (DEGs), such as antigen processing and presentation, nucleotide-binding and oligomerization domain (NOD)-like receptor signaling pathway, estrogen signaling pathway, progesterone-mediated oocyte maturation, MAPK signaling pathway, D-Glutamine, and D-glutamate metabolism. 

**Figure 5:**
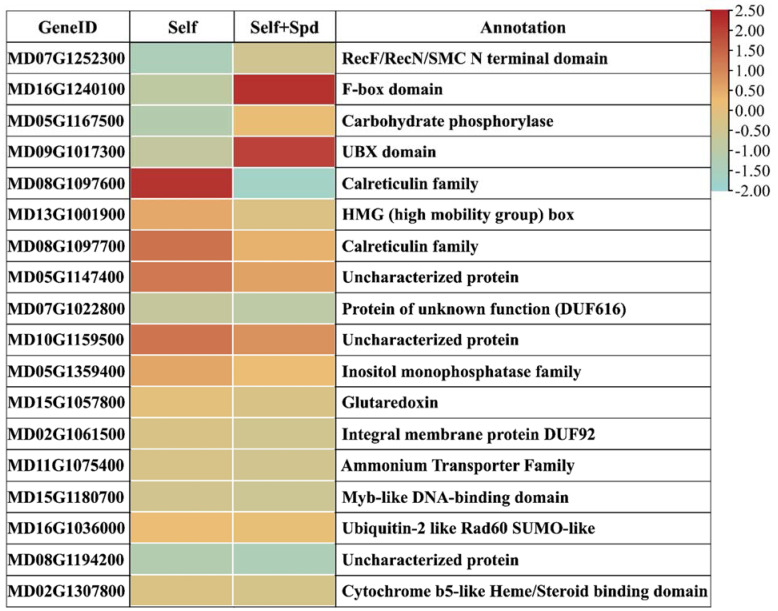
Heat map representing the differentially expressed transcripts between pollen tubes treated with self-S-RNase and pollen tubes treated with self-S-RNase and added 0.25 exogenous spermidine. Values are means of three biological replicates. Heatmap of log transformed FPKM values of differentially expressed transcripts differenced treated.

**Figure 6:**
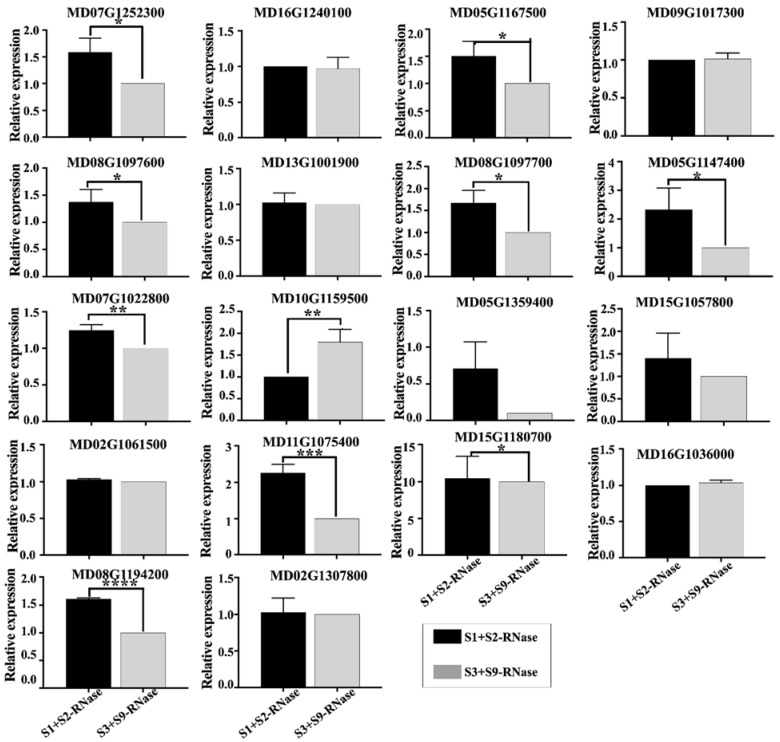
qRT-PCR analysis of gene treated pollen with self-S-RNase and non-self-S-RNase. Data are the means ± SD of three independent experiments. Asterisks indicate significant differences. *, *p* < 0.05; **, *p* < 0.01; ***, *p* < 0.001; ****, *p* < 0.0001 by *t* test.

To analyze the functions of the genes differentially expressed in response to spermidine treated, we identified DEGs for Fold Change > 2; four genes were upregulated and 14 genes were down-regulated (Figure 5). The RNA-seq and RT-qPCR data produced similar patterns of expression, thus confirming the reliability of the expression profiles obtained from RNA-Seq (Figure S3). By qRT-PCR, we found that there were significant differences in the expression of ten genes under self-incompatibility and non-self-incompatibility (Figure 6). Additionally, we found that the expression of eight genes was higher under self-S-RNase treatment than under non-self-S-RNase treatment, which was the same as that after spermidine treatment. These genes may be involved in spermidine breaking self-incompatibility.

## 4. Discussion

In apple, the self-incompatibility reaction is mediated by S-RNase. When the self-S-RNase secreted enters the pollen tube, it can cause a series of changes in the pollen tube, and the growth of the pollen tube is inhibited [25]. The same phenomenon was also found in our study. When we added the purified self-S-RNase in vitro to the hydrated pollen medium, the elongation of pollen was inhibited, while the elongation of pollen treated with non-self-S-RNase was weakly inhibited (Figure S1). Polyamines are very important physiologically active substances, which play an important role in regulating growth and development and resisting biological and abiotic stresses during plant growth [26]. In potato, it was found that low temperature could increase the content of putrescine [27]. In rice, it was found that the content of free putrescine and spermidine in the water stressed leaves, and spermine contents were higher than those of polyamines in rice under normal water [11]. S-RNase is also a kind of stress for pollen tube growth, which can trigger the plant defense response [28]. Whether self S-RNase can cause the change of polyamine content in apple pollen tube has not been studied. Therefore, we treated pollen tubes with self-S-RNase purified in vitro, compared with pollen treated with non-self-S-RNase and buffer. It was found that the content of putrescine, spermidine, and spermine in the pollen tubes increased first and then decreased after S-RNase treatment. When the difference in pollen tube germination length was the largest, the polyamine content after self-S-RNase treatment was lower than that of the control.

In citrus, it was found that the expression of ADC, SAMDC, SPDS, SPMS, DAO, and PAO increased after being induced by salt stress [29]. We also found a similar phenomenon in our study. When pollen was treated with self-S-RNase, the expression of SAMDC, DAO, and PAO increased significantly after 90 min of self-S-RNase treatment compared with that treated with non-self-S-RNase (Figure 2B). However, by determining the polyamine content, it was found that the polyamine content in the pollen tube decreased under the induction of self-S-RNase (Figure 1B). We found that the expression of SAMDC increased, but the polyamine content decreased, suggesting that the enzyme activity of SAMDC may be relatively low. The increased expression of diamine oxidase (DAO) and polyamine oxidase (PAO) may be the main reason for the reduction of polyamine content in pollen tubes caused by self-S-RNase. In order to find the key gene involved in polyamine metabolism in the self-incompatibility reaction, we silenced all the DAO and PAO family genes induced by self-S-RNase with antisense oligonucleosides. We found that only after silencing *MdPAO6*, was the inhibition of self-S-RNase on pollen tube growth significantly alleviated, while the accumulation of spermidine increased significantly (Figure 3C). Therefore, we speculate that the content of spermidine plays an important role in the elongation and growth of the pollen tube in the self-incompatibility reaction. In the study of *Arabidopsis*, it was found that the pollen tube growth of *Arabidopsis* mutant *Atpao3* was inhibited [19], which also confirmed that polyamine oxidase played an important role in the elongation and growth of the pollen tube.

In plants, putrescine, spermidine, and spermine play a role in growth, disease resistance, and reproduction. It was found that the application of spermidine in vitro during flower bud differentiation can significantly improve the flowering rate of ‘Fuji’ apple [30]. In *Arabidopsis*, it was found that the addition of a low concentration of spermidine in the pollen medium could promote the elongation of the pollen tube [9]. To study whether polyamines can improve the ability of pollen to resist S-RNase, we added different concentrations of polyamines to pollen culture medium, and then added self-S-RNase, non-self-S-RNase, and buffer control. We found that certain concentrations of spermidine and spermine could promote the growth of the pollen tube under self-incompatibility, the work found that the most suitable concentration to promote the growth of the pollen tube was 0.25 mmol/L (Figure 4B,D), and the same effect was found on observation of pollen tube fluorescence in the style (Figure 4E). The ‘Ralls Janet’ apple stigma was treated with 0.25 mmol/L spermidine, and then self-pollinated. After 72 h pollination, many pollen tubes grew to the bottom of the style. To study how spermidine helps pollen tubes resist the toxicity of self-S-RNase, we set up two self-S-RNase treated pollens, one with 0.25 mmol/L spermidine and the other without spermidine as control, and then conducted transcriptome analysis. To further identify the genes that responded to self-incompatibility and spermidine treatment, we analyzed DEGs for Fold Change > 2 by qRT-PCR. We detected the expression changes of these genes after the pollen with self-S-RNase and non-self-S-RNase respectively. It was found that there were significant differences in the expression of ten genes. Spermidine treatment can make pollen tubes break the self-incompatibility reaction caused by self-S-RNase, so that pollen tubes can grow normally. Therefore, the expression after spermidine treatment should be consistent with the expression trend of pollen tubes treated with non-self-S-RNase. Through this principle, we found that the expression of seven genes was high under self-S-RNase treatment and low under non-self-S-RNase treatment. We predicted the function of these genes; it was found that three of these genes are related to calcium ions, namely MD08G1097600, MD08G1097700, and MD05G1147400. MD08G1097600 and MD08G1097700 are calreticulin, as shown in Figure 5. Calreticulin is found in *Petunia* and plays an important role in maintaining the gradient of calcium ions at the tip of the pollen tube [31]. Previous studies in *Papaver* found that self-incompatibility can break the gradient of calcium ions at the tip of the pollen tube [32], and E-helix and F-helix hand protein can bind calcium ions to participate in calcium signal transduction. Therefore, we speculate that spermidine may regulate the expression of calreticulin or calcium binding protein, to adjust the change of the calcium concentration gradient after self-S-RNase treatment, and finally make the pollen tube grow normally. This may also explain why pollen tube growth is inhibited when pollen is treated with a high concentration of polyamines. It may be that excessive polyamines stimulate the changes of calmodulin and calcium binding protein gene expression, thus breaking the balance of calcium gradient at the pollen tube tip. The relationship between polyamines and calcium ions in the process of self-incompatibility is worthy of our study.

## 5. Conclusions

In this study, self-S-RNase treatment caused an increase in *MdPAO6* expression, which led to a decrease in spermidine content in pollen tubes. Silencing of *MdPAO6* in the pollen tube resulted in an increase of spermidine, thus the ability to resist self-S-RNase was improved. Treatment with 0.25 mmol/L spermidine in vitro was helpful in improving the ability of pollen tubes to resist self-S-RNase and promote pollen tube elongation. This may be due to the change in the amount of gene expression that regulates calcium ion concentration caused by spermidine treatment. This study has expanded our knowledge of the relationship between polyamines and self-incompatibility and provides a new strategy to break the self-incompatibility of apple pollen.

## Data Availability

All relevant data are included in the manuscript and Supplementary Files. RNA-seq data have uploaded to NCBI (SRA: PRJNA777758).

## Supplementary Materials

The following are available online at https://www.mdpi.com/article/10.3390/genes12111797/s1, Figure S1: Pollen tube length after treatment with self or non-self S-RNase, Figure S2: The heat map representation of genes involved in the polyamine metabolic pathway, Figure S3: Relative expression of selected DEGs in pollen tubes treated with self-S-RNase and in pollen tubes treated with self-S-RNase with added 0.25 exogenous spermidine, Table S1: Polyamine metabolism pathway gene information, Table S2: Primers used in this study, Table S3: RNA-Seq data statistics. Table S4. Oligodeoxynucleotide primer.
