# Supplementary material for: Polyamines Involved in Regulating Self-Incompatibility in Apple
Source: Genes (Basel). 2021 Nov 15;12(11):1797. doi: 10.3390/genes12111797 (PMC8620888; doi:10.3390/genes12111797)
Supplement: Supplementary file 1 [file genes-12-01797-s001.zip › genes-1441952-supplementary.pdf]

Table S1. Polyamine metabolism pathway gene information.

Gene Name	Gene ID	Position	Amino Acids	Isoelectric Point	Molecular Weight (Da)
MdSPDS1	MD13G1090400	Chr13:6387224…6390612	336	4.94	36437.61
MdSPDS2	MD16G1091000	Chr16:6320235…6324893	336	3.79	36391.61
MdSPDS3	MD14G1183600	Chr14:27610814…27615281	352	5.41	38934.7
MdSPDS4	MD06G1177500	Chr06:31725776…31729337	361	4.98	39431.57
MdSPMS1	MD01G1221000	Chr01:31308216…31313091	373	6.03	40938.89
MdSPMS2	MD01G1221200	Chr01:31336992…31340878	225	6.23	24714.58
MdSPMS3	MD07G1291100	Chr07:35184345…35189215	370	5.83	40578.49
MdSPMS4	MD07G1292400	Chr07:35363889…35366550	281	5.77	31277.23
MdODC1	MD05G1034000	Chr05:5447978…5449255	426	6.37	46450.28
MdODC2	MD05G1036600	Chr05:5889578…5890842	381	6.19	41568.71
MdSAMDC1	MD17G1070300	Chr17:5683437…5684610	375	4.79	40778.10
MdSAMDC2	MD09G1079800	Chr09:5601960…5603351	375	4.92	40674.04
MdSAMDC3	MD13G1178800	Chr13:14898409…14899476	356	6.15	39565.88
MdSAMDC4	MD16G1180000	Chr16:15448962…15450029	356	5.51	39281.47
MdSAMDC5	MD13G1052600	Chr13:3728771…3731492	362	4.79	40186.13
MdSAMDC6	MD16G1057400	Chr16:4105994…4108192	359	4.91	39810.72
MdDAO1	MD10G1316500	Chr10:39996570…39998816	306	5.1	33870.52
MdDAO2	MD02G1107000	Chr02:8653792…8655869	308	5.15	34093.85
MdDAO3	MD15G1226200	Chr15:18372781…18375238	309	5.76	34452.51
MdDAO4	MD02G1106500	Chr02:8615935…8617645	308	5.29	34110.75

MdDAO5	MD15G1226300	Chr15:18382523…18383249	177	5.96	19640.51
MdDAO6	MD15G1226400	Chr15:18394302…18395455	214	5.11	23347.39
MdDAO7	MD05G1342700	Chr05:46304166…46304772	73	7.84	8183.54
MdPAO1	MD02G1079400	Chr02:6259023…6260699	558	5.77	60905.58
MdPAO2	MD02G1306200	Chr02:35927595…35934510	361	5.62	40482.34
MdPAO3	MD09G1145900	Chr09:11357651…11361539	490	5.54	54054.96
MdPAO4	MD09G1280300	Chr09:35705060…35711858	491	5.51	54474.46
MdPAO5	MD15G1206900	Chr15:16460347…16462697	572	5.41	62996.78
MdPAO6	MD17G1287500	Chr17:34673835…34679547	499	5.60	55132.32
MdADC1	MD10G1062500	Chr10:8507601…8509960	731	5.23	78514.12
MdADC2	MD05G1055300	Chr05:9464512…9466896	729	5.26	77994.33

Table S2. Primers used in this study.

Gene ID		Forward sequence 5´---->3´	Reverse 5´---->3´
MD05G1034000	MdODC1	TGTAAGCCCCTCAAGGATAGTCT	ATGGCATCTTTTTATCTTGTTAAGTTC
MD05G1036600	MdODC2	CCAATCCATGCAAGGTCCTC	TACTATTGCTCCACGGTACACTTGA
MD10G1316500	MdDAO1	ATGGGAGATGGTGAGGGC	CTTCATCTCTGCCATCAGACTCAA
MD02G1107000	MdDAO2	GGAGTTCGGAAGGCTCGTAGT	GGAAATTTTTTCAGATCAATGGCTG
MD15G1226200	MdDAO3	GAAGAAGCAGAACAAAGATTCAC	ACATGAACAGCCTGAGGTGAGG
MD02G1106500	MdDAO4	AGTTTCGATCTCTTCATTCCAACGT	GTTTGCGGAGAGTCTTAGCTTTC
MD15G1226300	MdDAO5	GGACCGAAGCAGGAAGTAGTAGTAC	GGGTGCGTACGAATGCAACA
MD15G1226400	MdDAO6	TCCCACCAGATGTTAGCTTTATTAG	TAGGCAAGGATCAATGTCTACGAAG
MD05G1342700	MdDAO7	TCCCACCAGAGGACTTTTAGAGAGC	ATAAAGACTTGATAGTGTAAATGGG
MP02G1079400	MdPAO1	CCGAGATGAGCAGGTCAATGG	TCAAGTACCCTTTAGCAATGGTAAT
MP02G1306200	MdPAO2	ACCCATTATTCCACAACCCATGG	TCCTTGATTTGTATAATCCGAATAC
MP09G1145900	MdPAO3	CTCTTTGCTCAAATGGTGAGAAGA	AAGTCTATCTCGAGACTCCAACAGC
MP09G1280300	MdPAO4	CTGATACGCCGCCTAGGACTTAC	CATTCGACATATGTACCATTGTAAC
MP15G1206900	MdPAO5	GGAATCCGGAGGAGAACTCTGA	AACTAGCAGCACTACCAGAATCATC
MP17G1287500	MdPAO6	GCTGTTAATTTTGTGATGTTGCAG	TAACATCCAAGAGAATTCAGGTCCG
MD17G1070300	MdSAMDC1	CTTCAAACCAGCTGAGTTCTCTG	CTTGGAGACTGCGAACCAGAA
MD09G1079800	MdSAMDC2	GCTTTGTCAAGGACGACAGCG	CTAGATCTTTGCCAGACCTATTTCT
MD13G1178800	MdSAMDC3	ACCGTAAAAATGTAAGGTCGTCGT	CGAAGAGGCTGGACTCGGATA
MD16G1180000	MdSAMDC4	TGGCAAAGAGATGACGGCGA	AGCTAGCGTAGCTGTACCCGTC

MD13G1052600	MdSAMDC5	GAATCGAGCTCAGCCACAACA	TAACTGAAGCCATCTTCTGGTGTG
MD16G1057400	MdSAMDC6	TGCCGAATCAGCAAGCTTGA	TTGGGTTTTGTAGAATACAGAAGCC
MD10G1062500	MdADC1	CGGCGACCATCACCACCA	AGGAATTGACGGTGAAGTAGG
MD05G1055300	MdADC2	ATTACGATGGATCCAAATCTAGTGA	CACAAGCACTGGAAGAAATAGCCTC
MD13G1090400	MdSPDS1	TCTGTATAGGGGGAAATCAGAGTTC	GAGGTAGATGTGCTATCATCTCCTG
MD16G1091000	MdSPDS2	AGTTGTGGTGGTCAGAAATGGC	CCCTGGCCATATCGGACAAT
MD14G1183600	MdSPDS3	AGTTGTGGTGGTCAGAAATGGC	CCCTGGCCATATCGGACAAT
MD06G1177500	MdSPDS4	CCAGAGTGCAAGTACGCATAGATG	CCAGGAAGTCCTTATCAGAAAGTTC
MD01G1221000	MdSPMS1	GAGATAGATAAGATGGTTATAGATGTAAG	CGACAGGGTCTGACGAATCAAC
MD01G1221200	MdSPMS2	TAAGACAGATGCTTTAACTTGGTGC	TTCTTCTCCGTCTCCAGTCTCTAAC
MD07G1291100	MdSPMS3	AGTGGGAGAGGGAGAGTGCGAG	GAGAGAAGCAGAAACCAGAGATGTA
MD07G1292400	MdSPMS4	TCTGTATAGGGGGAAATCAGAGTTC	ATTCATCTTTCTCACTCAGCTGGAC
MD07G1252300		AACAGCTCAGTGCCCTATTAACCAG	GCATGTAACTTTTCTCGGTAAGCTCT
MD16G1240100		TCTCACTACCAAGTCGTCAGCGTTC	AAAGCACGCCATTTTCAAACACC
MD05G1167500		AAACCAATGGGATTACTCCTCGTC	CTGTGCCAAACGCTTCTTATTAGCC
MD09G1017300		CACTGCAGAAGATTATTATCGGGTT	ACCAAGCTCTGAACTGTTTCCGAAG
MD08G1097600		TCCGATGGACTCTCAAGCTACCAG	CAACCACGACAGAGACGATAACACC
MD13G1001900		AGGACAAAGATCCAAATGCACCCAA	GCAGCTTTATCCACGTAGGGCTTC
MD08G1097700		GAGCACCATCTCAAGGACCCTC	ATGCAGGCTCAAAATCTTTATTCGAC
MD05G1147400		GCGAATGTGGATCTCTTCGATGCGTA	CGCAAGGACCTGTTTGGGTGACCAGA
MD07G1022800		TTGGCGTTCAAACTCTGTACTTGCT	AGCCTTCTTTCCGTTGAACCTC
MD10G1159500		TTGAGATACCACAACAACCGATG	CCGTCCTAATGTACTTTTGACCGT
MD05G1359400		ATTTGGTCACGGAAACTGATAAGGCAT	TCCATCTACGGGATCAACTAGCCAT
MD15G1057800		TGTCATCATCTTCAGCCGAAACTCC	CTATTTCTCCCAGAGGGCACCGA
MD02G1061500		TCACGACTCTTAAGCCTGTTCGGA	CGGCAAGAGTAGAAATAGGTATCACCA
MD11G1075400		GTCAATTGCTGAGCGTACCCAA	CCAGAGCCTAACAACAAGTTACTCG
MD15G1180700		AGCTACTCCATGCTCCTCAAATC	TTCAAGCTTCTGAGTCTGAGACTGG
MD16G1036000		GTGGTTGAGAAGGTTGACGAAC	GAGGTAACCAGTCCTCCTCTTCC
MD08G1194200		AGACCAGCTGACAACCACAGTTAATCACC	CCTTGCAAAGCATCGACCGGACT
MD02G1307800		AGGCAACCAGGATGGTTATGA	TCAGATTTCTTTTTCATGGATGATG

Table S3. RNA-Seq data statistics.

sample	Raw reads	Clean reads	total map	Raw bases	Clean bases	Error rate(%)	Q20(%)	Q30(%)	GC content(%)	rRNA Ratio(%)
self_1	43919476	43409928	40070210(92.307%)	6.59E+09	6.32E+09	0.0267	97.25	92.66	47.83	3.14
self_2	50139846	49574298	45794160(92.375%)	7.52E+09	7.22E+09	0.0266	97.28	92.74	47.83	3.18
self_3	47706736	47294282	43712577(92.427%)	7.16E+09	6.68E+09	0.0262	97.46	93.08	47.65	3.12
self_spd_1	32772994	32030298	29494914(92.084%)	4.92E+09	4.7E+09	0.0272	97.01	92.22	48.07	3.06
self_spd_2	45813024	44789416	41061263(91.676%)	6.87E+09	6.52E+09	0.0271	97.09	92.37	47.66	3.33
self_spd_3	40210794	39611208	36593596(92.382%)	6.03E+09	5.75E+09	0.0268	97.23	92.6	47.88	3.29

Table S4. Oligodeoxynucleotide primer.

	S-ODN	AS-ODN
MdDAO4	AAGCAGTGTGCGAGCAGATTG	CAATCTGCTCGCACACTGCTT
MdPAO3	AGCTATGCACTCTTTGATATGGATG	CATCCATATCAAAGAGTGCATAGCT
MdPAO4	CAAGGCTATGACCCAATAATAAAGG	CCTTTATTATTGGGTCATAGCCTTG
MdPAO6	GTCACTGATCTGCAACTTTTA	TAAAAGTTGCAGATCAGTGAC

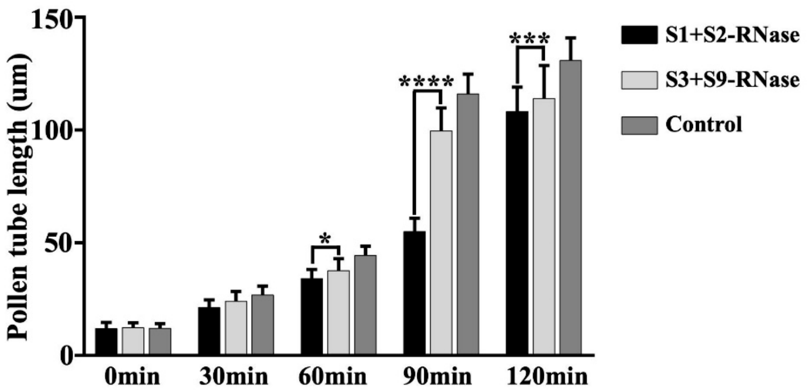

Figure S1. Pollen tube length after treated with self or non-self S-RNase.

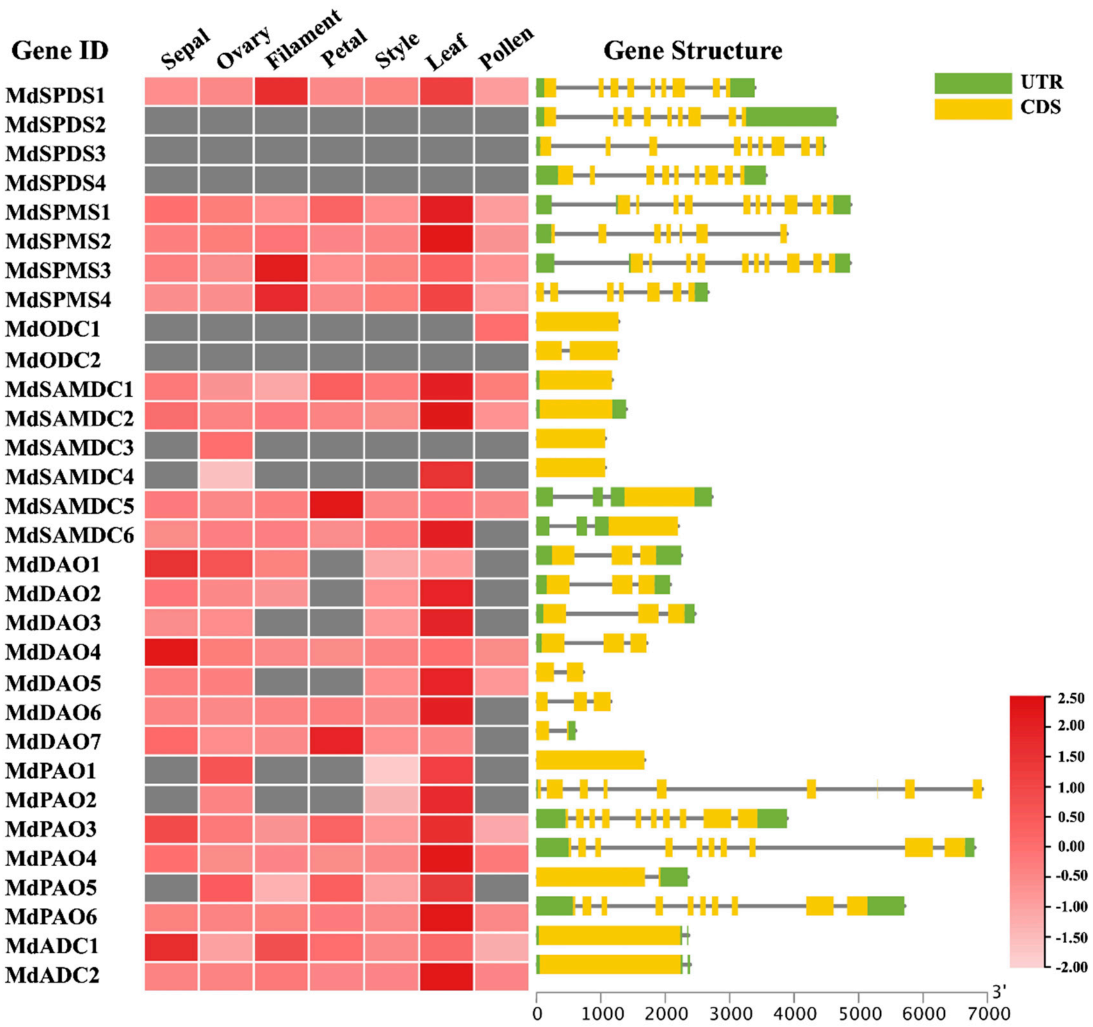

Figure S2. The heat map representation of genes involved in polyamine metabolic pathway. Gray color indicates tissues where the gene expression has not been measured.

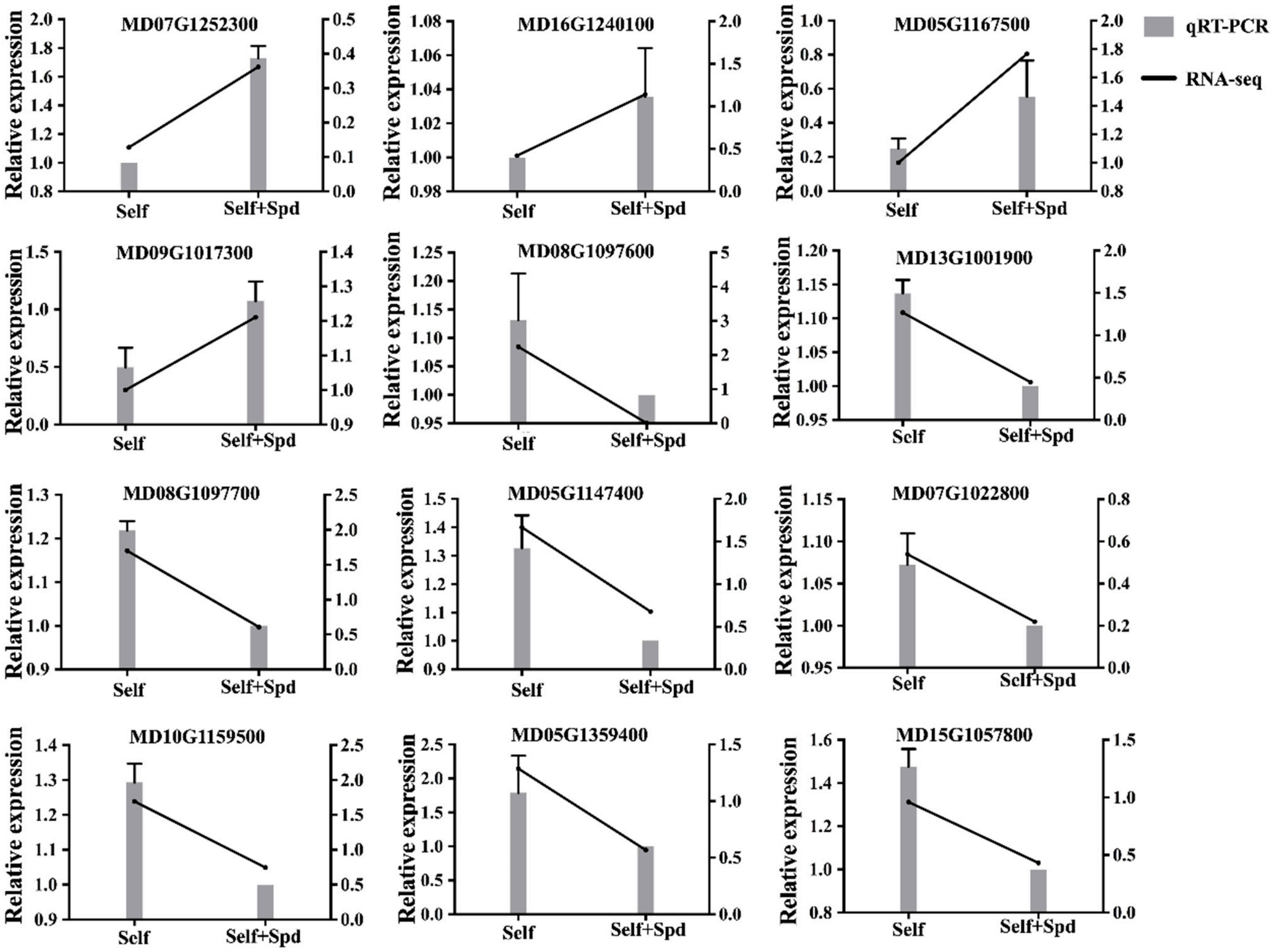

Figure S3. Relative expression of selected DEGs in pollen tubes treated with self-S-RNase and in pollen tubes treated with self-S-RNase added 0.25 exogenous spermidine. Relative expression levels were calculated CT value according to the $2^{-\Delta\Delta CT}$ method.
